# Signing below the dotted line: signature position as a marker of vulnerability for visuospatial processing difficulties

**DOI:** 10.1080/13554794.2013.860178

**Published:** 2013-12-07

**Authors:** Claire F Whitelock, Heather NAO Agyepong, Karalyn Patterson, Karen D Ersche

**Affiliations:** ^a^ Department of Psychiatry and Behavioural and Clinical Neuroscience Institute, University of Cambridge, Cambridge, UK; ^b^ Cognition and Brain Sciences Unit, Medical Research Council, Cambridge, UK

**Keywords:** handwriting, signature, neurological soft sign, visuospatial memory, drug dependence

## Abstract

Almost one-third of the participants in a neuropsychological study signed the consent form below the given line. The relationship between a signature position on or below the line and participants’ cognitive function was investigated. Fifty drug-dependent individuals, 50 of their siblings, and 50 unrelated control participants completed a battery of neuropsychological tests using the Cambridge Neuropsychological Test Automated Battery (CANTAB). Individuals signing below, rather than on, the line performed more poorly on tests of visuospatial memory, but no differently on other cognitive tests. Signature positioning may be a soft sign for impairment of the mechanisms involved in visuospatial memory.

A handwritten signature  is a distinctive way of indicating a person’s name. It is, of course, used as a means of identification, but a signature might also reveal more about a person than simply who he or she is. In clinical practice, handwritten information has long been of diagnostic value as it may convey information about the progression of certain disorders. In patients with Parkinson’s disease, for example, handwriting becomes smaller and more cramped as the disease develops: a phenomenon known as micrographia (Lewitt, 1983). A more subtle form of micrographia has also been observed in patients with obsessive-compulsive disorder (Mavrogiorgou et al., 2001), and handwriting difficulties also present in children with developmental coordination disorder (Rosenblum & Livneh-Zirinski, 2008). Changes in the shape and size of handwriting may therefore reflect impairments in the motor control that is required for writing, which is often associated with disorders such as the above (e.g., Mavrogiorgou et al., 2001). In addition, handwriting may provide indications about the writer’s cognitive functions (Phillips, Stelmach, & Teasdale, 1991). Measures of proficiency in sentence and name-writing, for example, seem to deteriorate in direct proportion to decreased cognitive functioning in aging and dementia (Ericsson, Forssell, Holmen, Viitanen, & Winblad, 1996). Although less a focus of research, handwritten signatures too have been associated with cognitive impairment. For example, patients with obsessive-compulsive disorder or Parkinson’s disease have difficulty signing their names as well as producing compositional writing or copying text (e.g., Ericsson et al., 1996; Mavrogiorgou et al., 2001).

In most research, handwriting ability is evaluated according to features such as letter height, letter and word spacing, speed of writing, and legibility. When signing a name, however, one is usually also expected to write in a particular way across the page: on the guideline, assuming one is provided. The positioning of handwriting or a signature on a page is therefore another typical characteristic of “normal” writing which may become impaired in certain individuals. The cognitive or perceptual processes involved in, and implications of, this aspect of writing seem to have been unexplored in the literature, despite the ease of assessing signature position quickly by the eye.

In a recent neuropsychological study of 150 individuals with and without stimulant drug dependence (Ersche et al., 2012a, 2012b), we observed that a number of participants across groups signed the study consent form below the signature line (see Figure 1(a)). We hypothesized that this behavior of signing below the line might be associated with cognitive impairment, given that other aspects of handwriting and signing performance have correlated with cognitive function in various other patient groups (e.g., Ericsson et al., 1996; Phillips et al., 1991). Handwriting and signing involve the coordination of a number of different processes, such as motor function, visuospatial perception, attention to the task, and kinesthestic feedback (Bonney, 1992; Feder & Majnemer, 2007; Harris & Livesey, 1992; Tucha & Lange, 2001). In the absence of previous literature to guide predictions, the domain of cognition associated with the observed atypical signature positioning seemed most likely to be visuospatial processing: when asked to sign on a line, one must perceive the location of the line and then act to position the signature on it.

**Figure 1.**
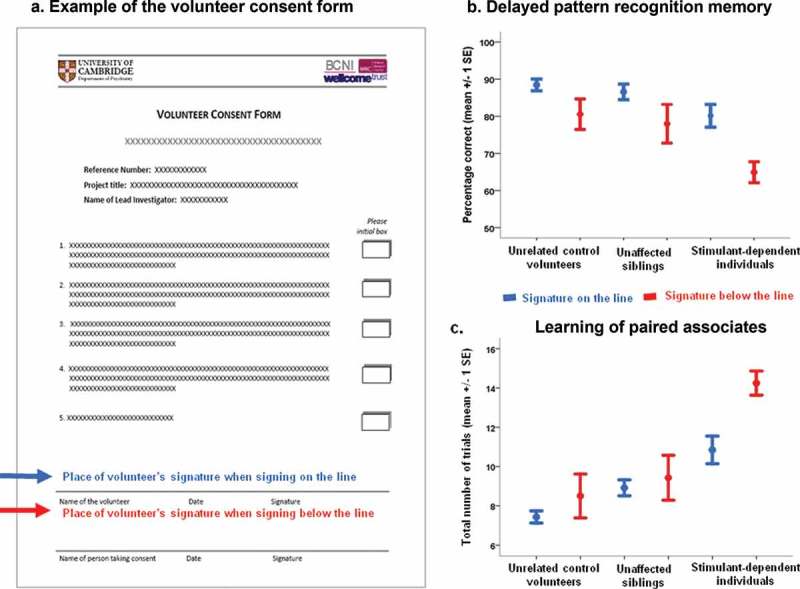
(a) An example of the volunteer consent form used in the present study, in which participants agree to five statements by initialing the boxes on the right-hand side, before giving consent to take part in the study by dating and signing the form at the bottom of the page. Seventy-one percent of volunteers signed the form on the signature line, while 29% placed their signatures below the signature line. In each of the three groups, there was a subgroup of individuals who signed below the signature line (12% control volunteers, 28% drug users’ siblings, and 48% drug-dependent volunteers). (b) Volunteers who signed below the line recognized significantly fewer visual patterns following a 25-min delay compared with volunteers who signed their consent above/on the signature line. (c) Volunteers who signed below the signature line needed significantly more trials to learn paired associates compared with their counterparts who signed on the line. [To view this figure in color, please see the online version of this journal.]

Participants completed a selection of tasks from the Cambridge Neuropsychological Test Automated Battery (CANTAB; www.camcog.com), a widely used neurocognitive assessment battery including tests of visuo-spatial memory, executive function, attention, and response control (Ersche et al., 2012a, 2012b). These tasks were selected on the basis of their association with dysfunction in drug dependence (see Ersche, Clark, London, Robbins, & Sahakian, 2006; Verdejo-Garcia, Lopez-Torrecillas, Gimenez, & Perez-Garcia, 2004; Vonmoos et al., 2013). In the present post hoc analysis, amongst these cognitive tasks, we hypothesized that inappropriate signature positioning would be associated with a selective deficit in visuospatial abilities measured by the Pattern Recognition Memory Test (PRM; Mehta, Sahakian, McKenna, & Robbins, 1999) and Paired Associates Learning Test (PAL; Sahakian et al., 1988), whilst performance on tests of executive or attentional function would be unrelated to signature position.

## Methods

Data were taken from the study reported by Ersche et al. (2012a, 2012b), which was approved by the Cambridge Research Ethics Committee (REC08/H0308/310; PI: KD Ersche). There were 150 participants in this study, aged between 18 and 55 years and able to read and write in English. One hundred of the participants comprised 50 biological sibling pairs; the remaining 50 were unrelated healthy volunteers. Within each sibling pair, one sibling met the criteria for dependence on stimulant drugs (94% cocaine, 6% amphetamines) and some for dependence on additional substances (54% opiates, 24% alcohol, and 8% cannabis), whereas the other sibling had no history of chronic drug or alcohol abuse. The unrelated healthy volunteers had no personal or family history of drug or alcohol dependence. The drug-taking experiences in both the siblings and control volunteers were minor, as reflected by very low scores on the Drug Abuse Screening Test (DAST-20; Gavin, Ross, & Skinner, 1989: siblings: 0.5 ± 1.1 standard deviation (SD), controls: 0.0 ± 0 SD) and the Alcohol Use Disorders Identification Test (AUDIT; Saunders, Aasland, Babor, de la Fuente, & Grant, 1993: siblings: 3.8 ± 4.5 SD, controls: 3.3 ± 2.3 SD). Participants were psychiatrically evaluated using the structured clinical interview for DSM-IV-TR (First, Spitzer, Gibbon, & Williams, 2002). Exclusionary criteria were a history of a psychotic disorder, a neurological illness, a neurodevelopmental disorder, or a traumatic head injury.

All except five drug-dependent participants were actively using stimulant drugs, as verified by urine screen prior to testing, and all urine screens provided by siblings and control volunteers were negative for drugs of abuse. We verified that participants were not under the influence of drugs or alcohol at the time of testing by checking for signs of acute drug intoxication or withdrawal.

Participants were assessed at the Wellcome Trust Clinical Research Facility, Addenbrooke’s Hospital, Cambridge, United Kingdom, and written informed consent was obtained prior to study enrolment. Each participant then performed the same battery of CANTAB neuropsychological tests in a fixed order at the same time of the day (see Table 1), as well as a variety of other measures, including the National Adult Reading Test (NART; Nelson, 1982) as an estimate of verbal IQ, and the Beck Depression Inventory (BDI-II; Beck, Steer, & Brown, 1996) to record depressive mood.

**Table 1.** Summary of neuropsychological tests from the CANTAB battery (www.camcog.com).

Domains	Task descriptions	Key outcome measures
**Visual memory battery**		
PRM	A two-choice test of abstract visual pattern recognition memory (Mehta et al., 1999).	Percentage correct (*immediately after presentation and following a 25-min delay*)
PAL	A test of episodic memory which involves the learning of spatial locations of geometric visual patterns (Sahakian et al., 1988).	Total trials (*number of total trials needed to learn paired associates*)
		First trial memory score (*the sum of patterns correctly located after first presentation*)
**Executive function battery**		
SWM	A self-ordered search task involving a search through a spatial array of colored boxes for tokens, without returning to a box which had already contained a token (Owen, Downes, Sahakian, Polkey, & Robbins, 1990).	Total errors
OTS	A spatial planning test involving planning a sequence of moves to achieve a goal arrangement of colored balls without moving the balls (Owen, Sahakian, Semple, Polkey, & Robbins, 1995).	Mean attempts to solve planning problems at varying levels of difficulty
**Attention battery**		
RTI	A reaction time task which uses a procedure to separate response latency from movement time (Sahakian et al., 1993).	Accuracy score
RVIP	A test of sustained attention which involves the detection of a target sequence of three digits in a sequential presentation of single digits presented at a rate of 100 digits per min in a pseudo-random order (Park et al., 1994).	Target sensitivity A (*measure of discriminability between signal and noise*)
**Response control**		
STOP	A test of response inhibition which uses staircase functions to generate an estimate of STOP reaction time (SSRT; Logan, Schachar, & Tannock, 1997).	SSRT (*measure of response inhibition*)

### Statistical analysis of cognitive data

Statistical analyses were performed using the Statistical Package for the Social Sciences version 20 (SPSS, IBM). All tests were two-tailed, and an effect was deemed significant at *p *< 0.05. To explore group differences in demographics and cognitive performance, analyses of co-variance (ANCOVA) were used with group (three levels: drug users, siblings, controls) and signature (two levels: on the line, below the line) as between-subject factors. Chi-square or Fisher’s exact tests were used for the analysis of categorical data. For tasks with more than one stage or level of difficulty, repeated-measures ANCOVA models were employed. Where the assumption of sphericity was violated in repeated-measures ANCOVA, within-subjects degrees of freedom were adjusted using the Greenhouse–Geisser correction. The sex of the participant was included as a covariate in all analyses to control for the significant group differences in proportion of males/females. Age and the BDI-II were also included as covariates in the analysis of the cognitive data to control for age differences and for potentially confounding effects of dysphoric mood on cognitive performance (Hartlage, Alloy, Vazquez, & Dykman, 1993). For post hoc comparisons, the Dunn–Sidak correction was applied. Due to technical problems, Rapid Visual Information Processing (RVIP), One Touch Stockings of Cambridge (OTS), and stop-signal (STOP) data of one drug-dependent participant were unavailable.

## Results

### Demographic group differences

One-fifth of participants (30 individuals) signed below the signature line on the written informed consent form. A signature was considered “below the line” if the whole signature was placed below the given line (see Figure 1(a)). The tendency to place the signature below the line was unequally distributed across the three participant groups of drug-dependent individuals, their siblings, and healthy controls (Fisher’s exact *p *< 0.001). Almost half of the drug-dependent participants (48%; *N* = 24) provided their signature below the line, compared with 28% (*N* = 14) of their siblings and 12% (*N* = 6) of the control volunteers. In addition, although age between the three groups was well matched (*F*_2,143_ = 0.6, *p* > 0.5), participants who signed below the line were significantly older (mean: 36 years, ±8.1 SD) than their counterparts who signed on the line (mean: 32 years, ±8.1SD) (*F*_1,143_ = 7.1, *p *= 0.008). However, the sex distribution was not significantly different between the two signature types (*χ* ^2^ = 3.1, *p *> 0.05), but did differ between the three groups (*χ* ^2^ = 16.8, *p *< 0.001), as the drug-dependent group was male-dominated. Dysphoric mood did not significantly differ between the signature types (*F*_1,143_ = 1.3, *p *> 0.1) but did between the groups (*F*_2,143_ = 31.5, *p *< 0.001) because the drug-dependent individuals scored significantly higher on the BDI-II compared with the other two groups (both *p *< 0.001). Finally, no differences were found for intelligence levels or years of education between the signature types or the groups. To obviate any potential effects of recent drug use on the signature position, we also compared drug-dependent individuals who signed below or above the line with regard to the time elapsed since last they last used drugs, but no significant group difference emerged (*t*_47_ = –0.36, *p *= 0.724).

## Cognitive group differences

### Visuospatial memory

On the test of PRM, significant main effects of signature type (*F*_1,141_ = 10.2, *p *= 0.002) and group (*F*_2,141_ = 4.4, *p *= 0.014) were identified. Individuals who signed below the line recognized fewer patterns compared with their counterparts who signed on the line (*p *= 0.002), and drug-dependent participants also recognized fewer patterns compared with their siblings and unrelated healthy volunteers (both *p *< 0.05). All participants recognized more patterns immediately after presentation than following a 25-min delay, as reflected by a significant main effect of delay (*F*_1,141_ = 5.5, *p *= 0.021). There was also a significant delay-by-signature interaction (*F*_1,141_ = 8.2, *p *= 0.005), suggesting that poor recognition memory in individuals who signed below the line was exacerbated by delay (see Figure 1(b)). The delay-by-group interaction was not significant (*F*_2,141_ = 1.3, *p *> 0.1).

Significant differences between signature types (*F*_1,141_ = 5.9, *p *= 0.016) and groups (*F*_2,141_ = 12.0, *p *< 0.001) also emerged for the learning of paired associates, a test of visual memory and new learning. As shown in Figure 1(c), individuals signing below the line needed significantly more learning trials than their “on the line” counterparts (*p *= 0.016). Drug-dependent individuals also needed significantly more trials compared with their siblings and healthy volunteers (both *p *< 0.001), and were more affected by the level of task difficulty, as reflected by a significant difficulty-by-group interaction (*F*_3.5,252_ = 3.4, *p *= 0.013). Interestingly, memory for paired associates only differed between the groups (*F*_2,141_ = 7.1, *p *= 0.001) and not between signature types (F_1,141_ = 2.5, *p *> 0.1). Again, the group difference was due to drug-dependent individuals remembering significantly fewer paired associates than their siblings and unrelated healthy volunteers (both *p* < 0.005).

### Executive function

Performance on the spatial working memory (SWM) task differed between the groups (*F*_2,141_ = 4.8, *p *= 0.010) but not between signature types (*F*_1,141_ = 2.4, *p *> 0.1). Drug-dependent individuals exhibited significantly more errors compared with the other two groups (both *p *< 0.05). For mental planning (OTS), a similar picture emerged: a significant main effect of group (*F*_2,141_ = 16.5, *p *< 0.001) but not of signature type (F_1,141_ = 2.9, *p *> 0.05). The group effect was due to drug-dependent individuals needing more attempts to solve planning problems compared with their siblings and unrelated healthy volunteers (both *p *< 0.001). There was also a significant main effect of difficulty (*F*_2.5,141_ = 7.7, *p *< 0.001) and a significant difficulty-by-group interaction (*F*_4.9,141_ = 7.5, *p *< 0.001), indicating that planning performance in the groups was significantly modulated by the level of task difficulty.

### Attention

Response accuracy on the reaction time task (RIT) did not differ between signature types (*F*_1,141_ = 0.3, *p *> 0.5), but revealed a significant difference between groups (*F*_2,141_ = 4.8, *p *= 0.010): drug-dependent individuals were less accurate in responding than the other two groups (both *p *< 0.05). Neither the signature types (*F*_1,141_ = 2.7, *p *> 0.5) nor the groups (*F*_2,141_ = 1.8, *p *> 0.5) differed in terms of discrimination accuracy on the test of sustained attention.

### Response control

On the STOP task, which is a measure of inhibitory motor control, SSRT yielded no significant differences of signature type (*F*_1,141_ = 1.9, *p *> 0.1) or group (*F*_2,141_ = 1.7, *p *> 0.1). The overall probability of inhibition *p*(STOP) was >0.5, indicating that the algorithm (on the basis of which SSRTs were obtained) was appropriately calculated.

## Discussion

In our previous study, we investigated cognitive markers of vulnerability for the development of drug dependence by comparing cognitive function in three groups: 50 drug-dependent individuals, 50 of their unaffected biological siblings, and 50 unrelated healthy controls (Ersche et al., 2012a, 2012b). These participants provided informed consent in writing, and we noticed on completion of the study that (1) a number of these participants signed the consent form below, rather than (as expected) on the signature line provided on the form, and (2) the atypical-position signers were unequally distributed between the three groups. The aim of the present analysis was to investigate whether cognitive performance in these 150 individuals was a function of their signature position as well as group, hypothesizing that below-the-line signers would have significantly poorer performance on tasks of visuospatial function compared with their counterparts who signed on the line. This hypothesis was supported by the analysis, and the impairment profile was specific for visuospatial function, and not observed in tasks of executive function, attention, or response control. Individuals who signed below the line not only recognized fewer abstract visual patterns immediately after presentation and following a 25-min delay, they also needed significantly longer to learn the spatial locations of visual patterns during paired associate learning. Counter to hypothesis, however, performance in the immediate recall of the location of visual patterns was not impaired in the below-the-line subgroup.

We had predicted that signing below the line would be associated with impairment in all visuospatial tasks in the study, including immediate location memory, because visuospatial processing seemed the most likely candidate for explaining inappropriate signature positioning. Visuospatial memory does not seem such a ready explanation for signing below the indicated line, because signing on a line that is currently in the visual field does not require memory. It is, however, possible that participants signing below the line have a relatively subtle visuospatial deficit, which is unmasked by memory load in the delayed-response condition of the pattern recognition task, but that is not apparent in the simpler task of immediate responding. This interpretation is supported by the significant interaction between signature type and delay. The number of trials needed to learn meaningless associations between patterns and their locations in a display might also be sensitive to this subtle deficit, and the below-the-line signers did require significantly more learning trials in the PAL component of the cognitive battery.

As drug-dependent participants were more likely to sign below the line than their unaffected siblings, who in turn were more likely to do so than unrelated healthy volunteers, it is also possible that the subtle visuospatial deficit associated with signing in an abnormal position signals an underlying vulnerability in certain individuals, which is exacerbated by chronic drug use. For example, research in both animals and humans has shown that chronic drug use can impair cognitive functions such as attention, visual and working memory, decision-making, response-inhibition, and planning (Briand et al., 2008; Dalley et al., 2005; Ersche et al., 2006; Verdejo-Garcia et al., 2004; Vonmoos et al., 2013).

We were limited in the present study by its post hoc nature, and were therefore unable to assess the relationship between non-memory aspects of visuospatial function and signature positioning more directly and extensively. Further research investigating the relationship between cognition and signature position would be needed to replicate and expand upon the present results. Other visuospatial aspects of signatures, such as letter spacing, might also be studied for their association with visuospatial dysfunction.

Still, it seems unlikely that the present results are spurious: in the post hoc examination of an independent study which used the same neuropsychological tests, we also found that those signing below the line performed more poorly on the PRM task and in pattern location learning, but not in immediate pattern location (unpublished results). We therefore believe that this apparently robust result may be of interest to clinicians or researchers, for whom signatures of patients or participants are readily available through signed consent forms. On the basis of the present results, we can only conclude that signing below the line may be a soft sign for abnormal visuospatial learning and memory (and possibly other as yet unidentified measures of visuospatial function), which might be exacerbated by chronic drug use, in the manner of other cognitive functions (e.g., Ersche et al., 2006; Verdejo-Garcia et al., 2004).

## Funding

This work was funded by the Medical Research Council [grant number G0701497] and conducted within the Behavioural and Clinical Neuroscience Institute, University of Cambridge, United Kingdom, which is supported by a joint award from the MRC and the Wellcome Trust. Claire Whitelock and Heather Agyepong were both supported by the National Institute of Health Research (NIHR), and Karen Ersche was supported by the Medical Research Council.
